# Supplementary material for: Conversion of invisible metal-organic frameworks to luminescent perovskite nanocrystals for confidential information encryption and decryption
Source: Nat Commun. 2017 Oct 31;8:1138. doi: 10.1038/s41467-017-01248-2 (PMC5663915; doi:10.1038/s41467-017-01248-2)
Supplement: Supplementary file 1 — Supplementary Information [file 41467_2017_1248_MOESM1_ESM.pdf]

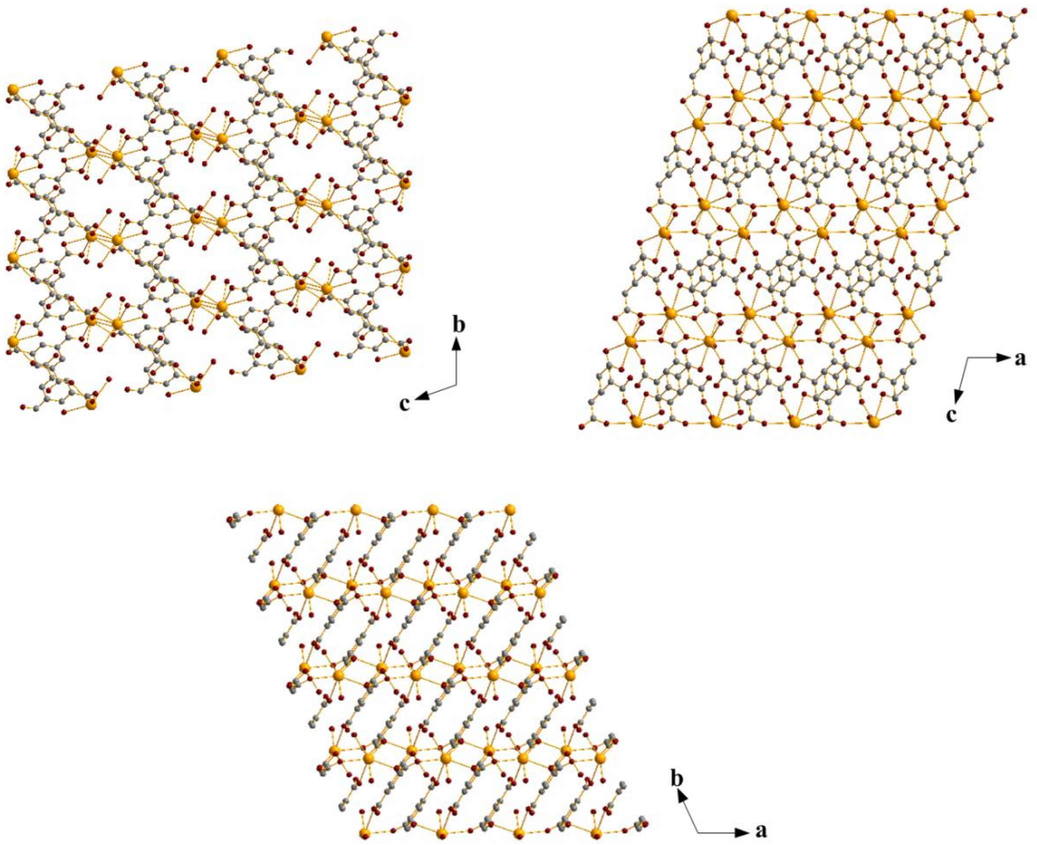

Supplementary Figure 1 Crystal structure of the Pb-MOF (Pb_2(1,3,5-HBTC)$_2$(H_2O)$_4$). H-atoms have been omitted for clarity; atom color scheme: Pb = yellow, C = gray, O = red.

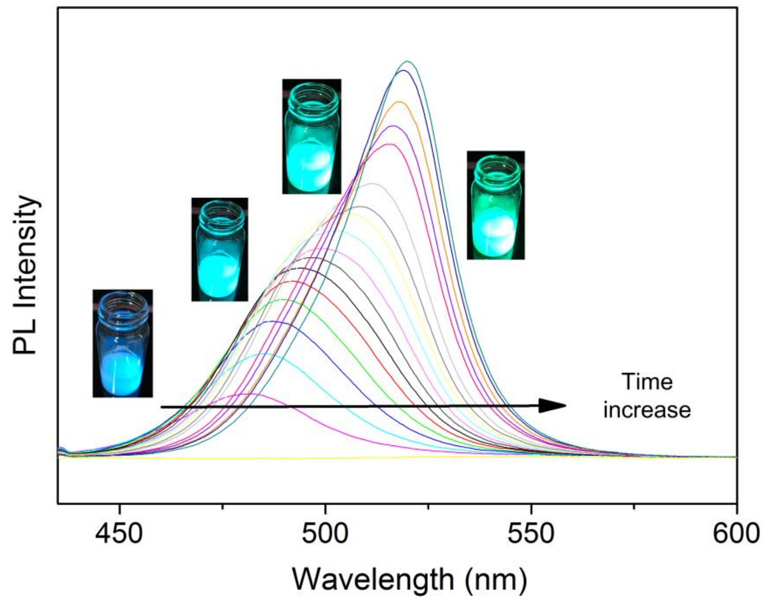

Supplementary Figure 2 The color and the PL spectra of the reactive system changed with time increase.

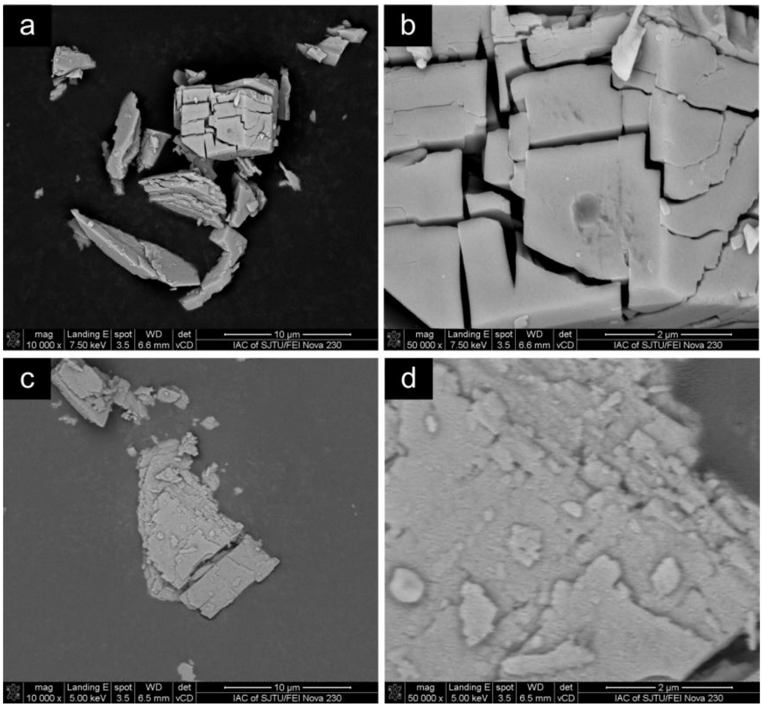

Supplementary Figure 3 SEM images of a, b) Pb-MOF and c, d) $MAPbBr_3$ NCs@Pb-MOF.

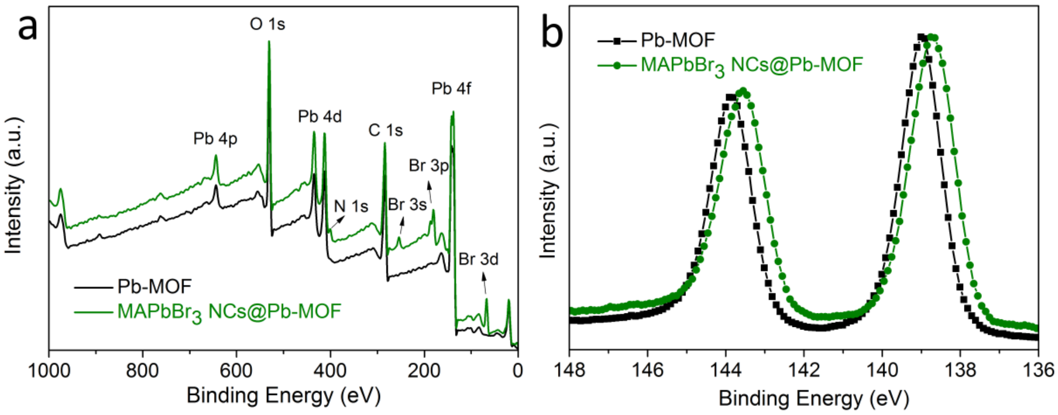

Supplementary Figure 4 XPS spectra of original the Pb-MOF and $MAPbBr_3$ NCs@Pb-MOF: a) the full-range spectra; b) Pb 4f spectra.

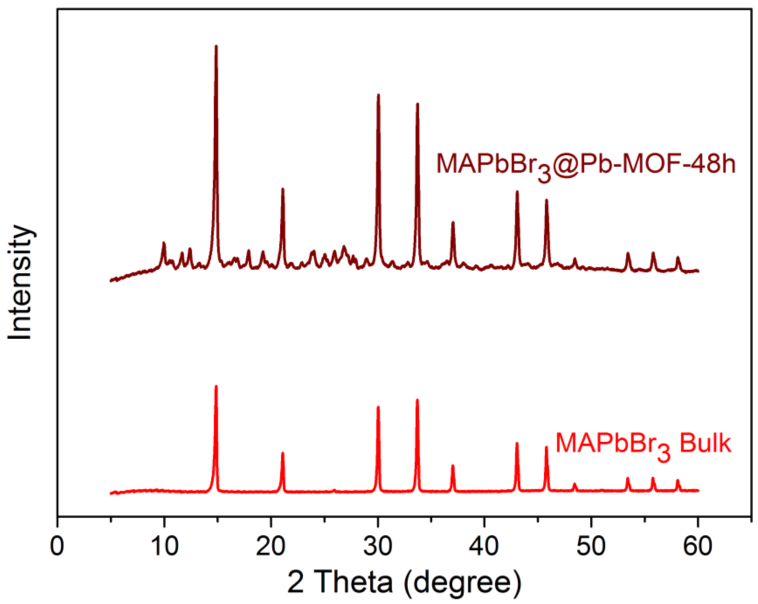

Supplementary Figure 5 XRD patterns of the $MAPbBr_3$@Pb-MOF powder after a long time (48 h) and high reactant concentration of MABr (20 times than used for luminescent $MAPbBr_3$ NCs@Pb-MOF) conversion process.

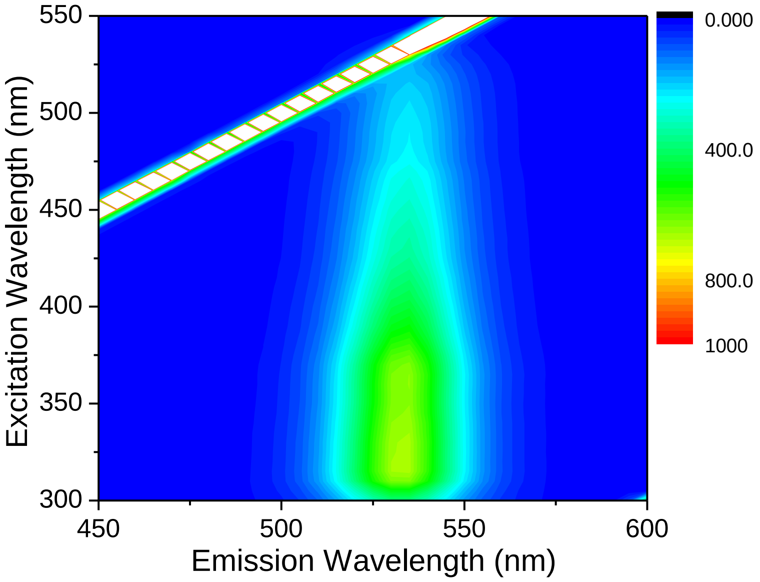

Supplementary Figure 6 EEM spectrum of the $MAPbBr_3$ NCs@Pb-MOF powder.

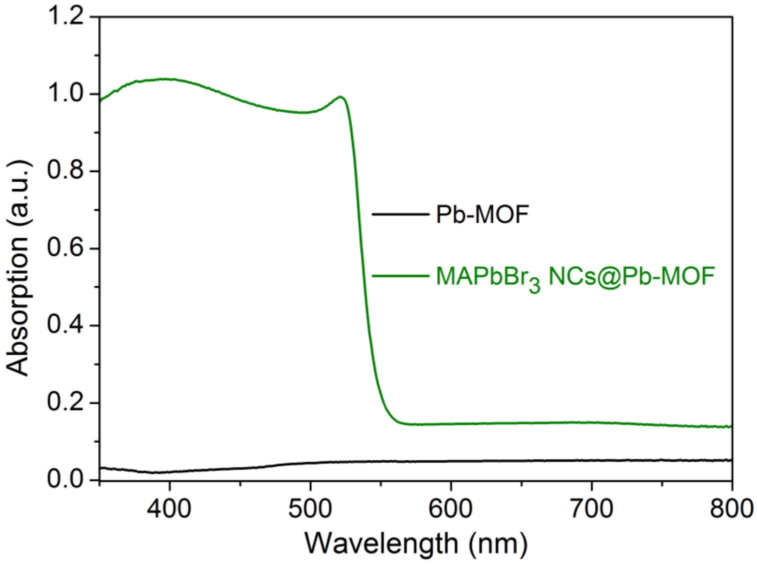

Supplementary Figure 7 UV-vis absorption spectra of Pb-MOF and $MAPbBr_3$ NCs@Pb-MOF.

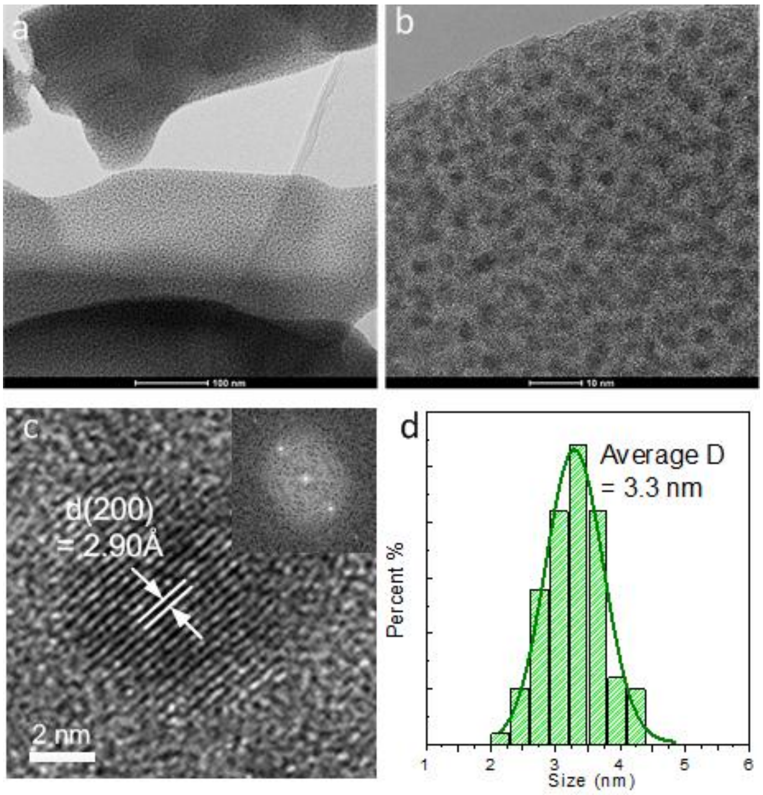

Supplementary Figure 8 TEM images (a, b), HRTEM image (c), and size distribution (d) of the $MAPbBr_3$ NCs@Pb-MOF sample without drying step.

To prove the quantum confinement phenomenon during the perovskite NCs' growth process, we conducted the TEM characterization of the powder sample before rinsing and drying. As shown in Supplementary Fig. 8, the small $MAPbBr_3$ QDs (~ 3.3 nm) are embedded in Pb-MOF matrix with good dispersion, from which the interplanar spacing of about 2.9 Å corresponding to the (200) crystal faces of the $MAPbBr_3$ crystal can be confirmed.[37]

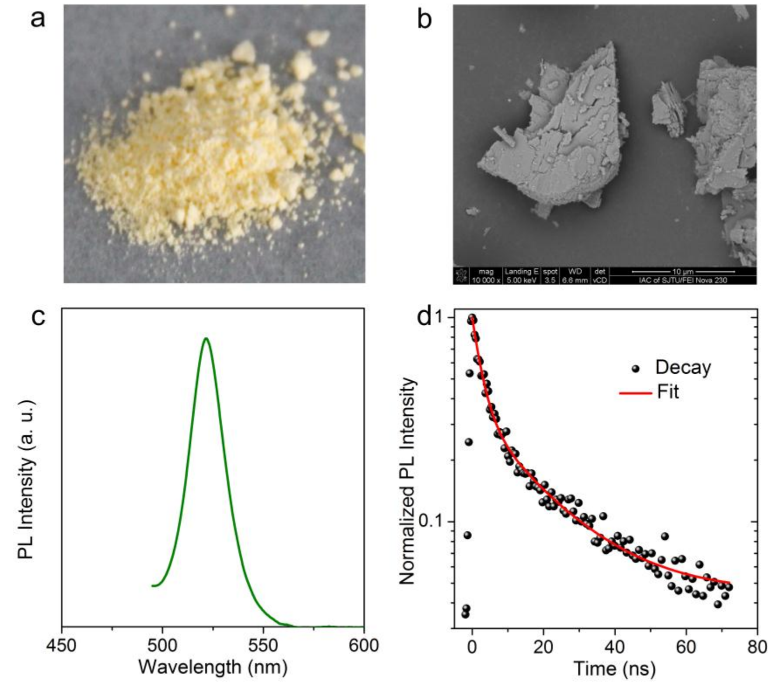

Supplementary Figure 9 Characterization of $CsPbBr_3$ NCs@Pb-MOF. a) The photograph, b) SEM image, c) PL spectrum and d) PL decay curve of the $CsPbBr_3$ NCs@Pb-MOF sample.

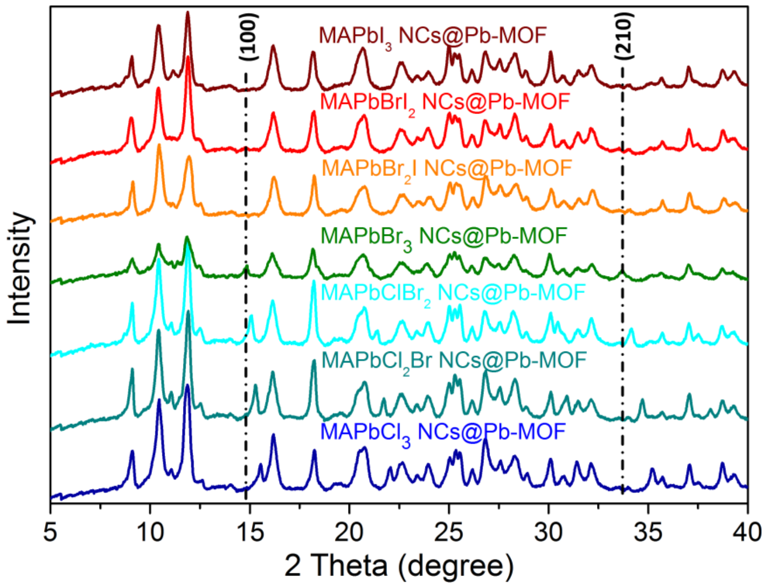

Supplementary Figure 10 XRD patterns of single and mix-halide $MAPbX_3$ NCs@Pb-MOF samples.

The peak intensities of these iodide-containing samples were relative lower compared with others, which may be ascribed to the fast degradation of the iodide-containing perovskite crystals in air.

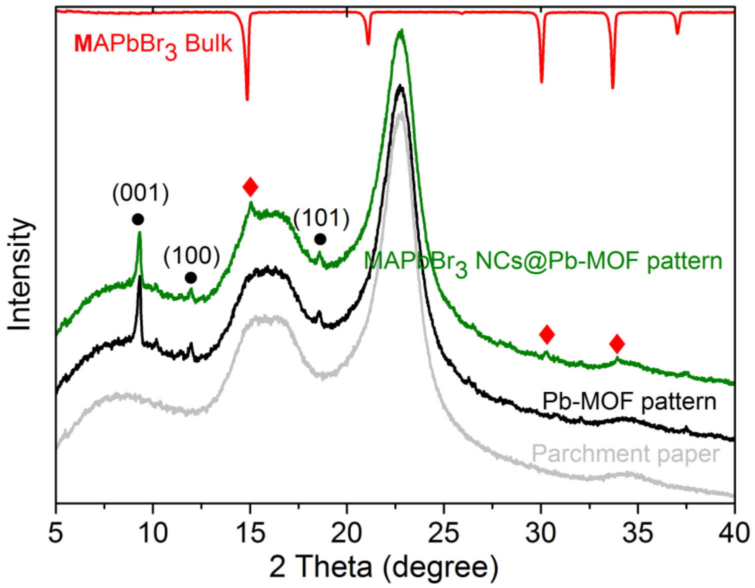

Supplementary Figure 11 XRD patterns of the parchment substrates with Pb-MOF and the $MAPbBr_3$ NCs@Pb-MOF patterns.

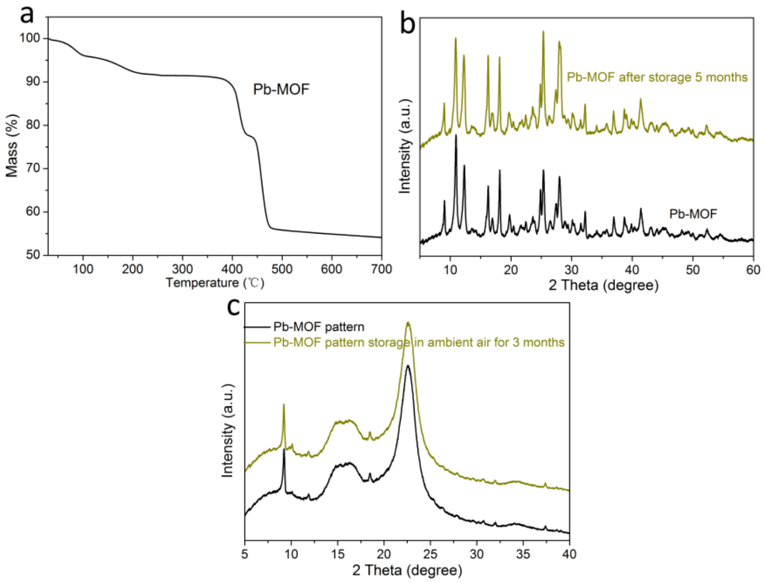

Supplementary Figure 12 Stability of the Pb-MOF powder and the Pb-MOF pattern: a) TG curve of the Pb-MOF powder, b) XRD patterns of the Pb-MOF powder before and after storage 5 months, c) XRD data of the printed Pb-MOF pattern after storage 3 months.

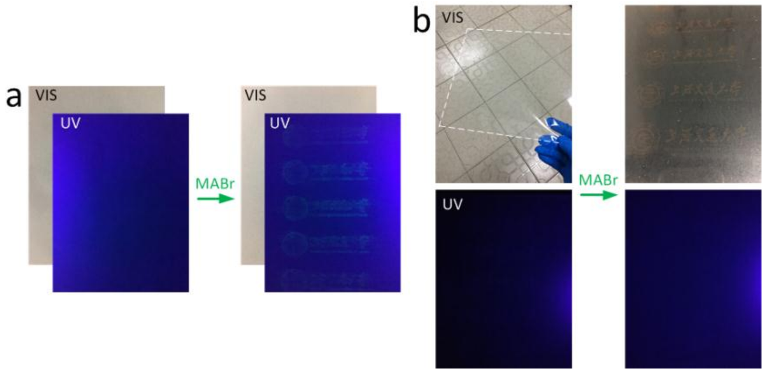

Supplementary Figure 13 Photographs of the printed patterns on parchment (a) and PET (b) substrate using Pb^{2+} ink before and after MABr treatment under ambient and UV light.

The Pb^{2+} ink composition was similar to the Pb-MOF ink except the absence of the H_3BTC linker. Subsequently, the parchment was used as the substrate for printing process. As shown in Supplementary Fig. 13a, the printed pattern was invisible. After the conversion with MABr, the fluorescent $MAPbBr_3$ NCs could be formed. This can be ascribed to the confinement effect of the textured structure of the parchment substrate. But the fluorescence seems to be relative dim and blurry compared with the $MAPbBr_3$ NCs@Pb-MOF pattern, making it difficult to identify the printed information. Actually, this phenomenon is not unexpected given that the absence of organic linker. In this case, because of the absence of any H_3BTC linker, the printed Pb^{2+} pattern cannot be fixed by the crystal framework, possibly making them easy to move around when the parchment substrate was contact with high affinity solvent (such as alcohol). To further verify the feasibility of the Pb^{2+} ink, we used the PET substrate. The results were shown in Supplementary Fig. 13b, it is obvious that the fluorescent perovskite NCs cannot be formed in this case. This can also be attributed to the absence of organic linker and crystal framework, which cannot restrict or confine the growth of the perovskite NCs on the smooth flat PET surface. From these results, we can demonstrate that the Pb^{2+} ink cannot be directly used for the information encryption and decryption application in our work.

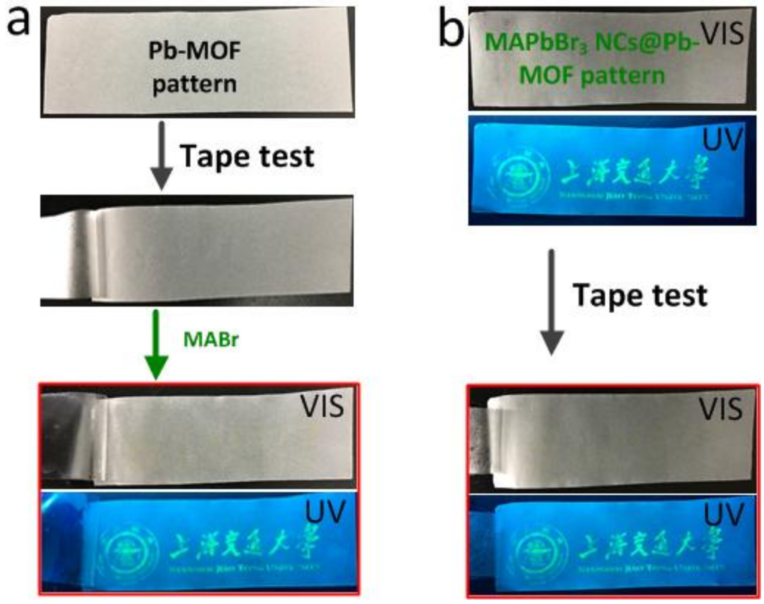

Supplementary Figure 14 Tape test results of printed Pb-MOF (a) and $MAPbBr_3$ NCs@Pb-MOF (b) pattern.

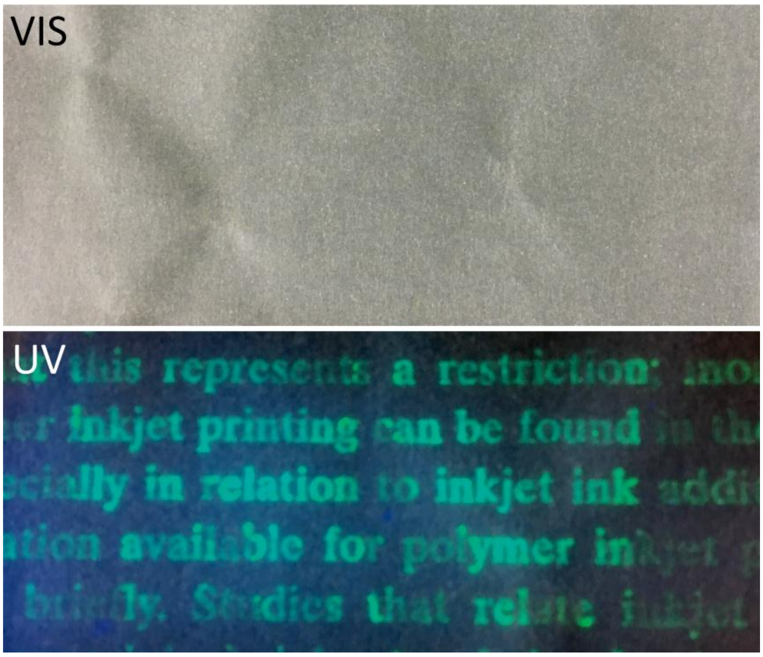

Supplementary Figure 15 Photographs of the $MAPbBr_3$ NCs@Pb-MOF patterns exposed in air for three months under ambient and UV light.

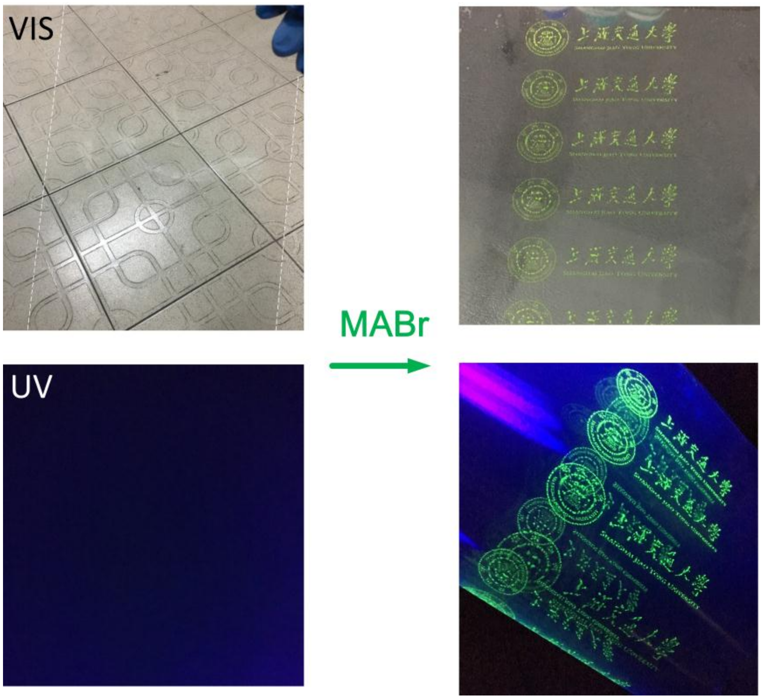

Supplementary Figure 16 Photographs of the transparent PET foils with Pb-MOF and the $MAPbBr_3$ NCs@Pb-MOF patterns under ambient and UV light.

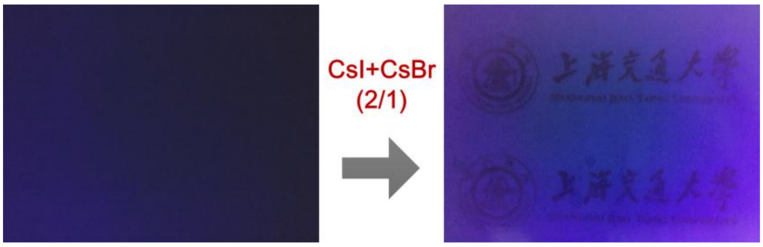

Supplementary Figure 17 Photographs of the parchment substrates with Pb-MOF and the $CsPbBr_3$ NCs@Pb-MOF patterns under UV light.

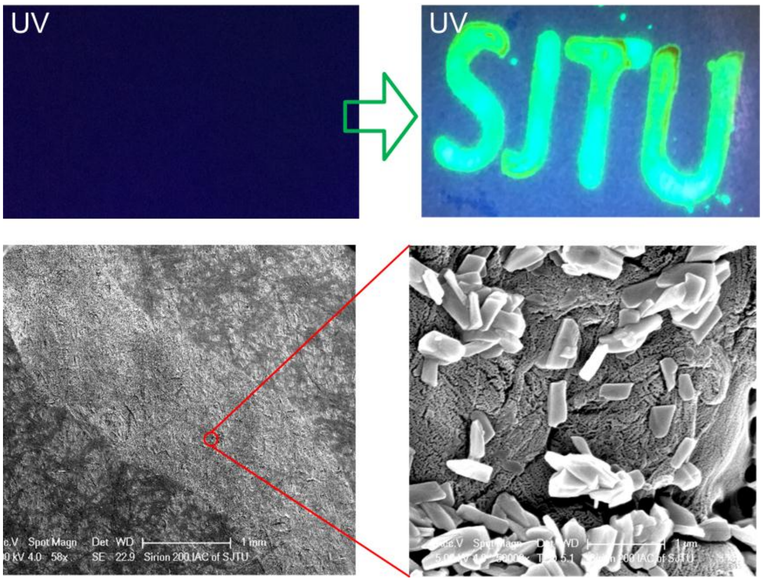

Supplementary Figure 18 Photographs of the parchment substrates with Pb-MOF pattern printed by contact printing technique (upper left) and corresponding $MAPbBr_3$ NCs@Pb-MOF pattern after conversion process by MABr (upper right); SEM images of the Pb-MOF pattern printed by contact printing technique (bottom).

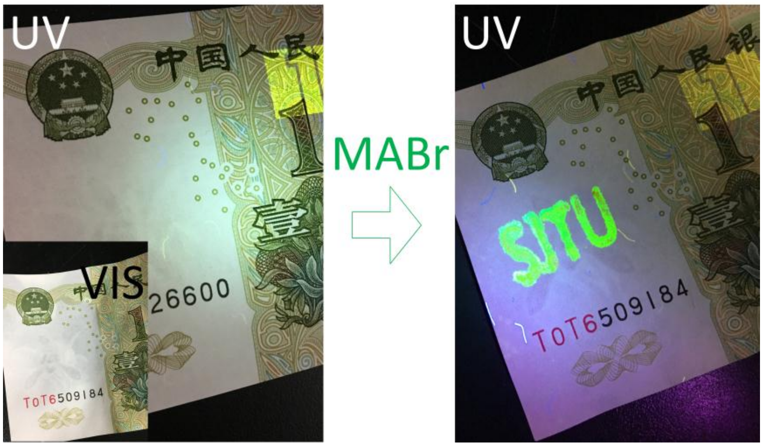

Supplementary Figure 19 Photographs of the banknotes (taken by Zhichun Li, one of the contributing authors) with Pb-MOF and the $MAPbBr_3$ NCs@Pb-MOF patterns printed by contact printing technique.

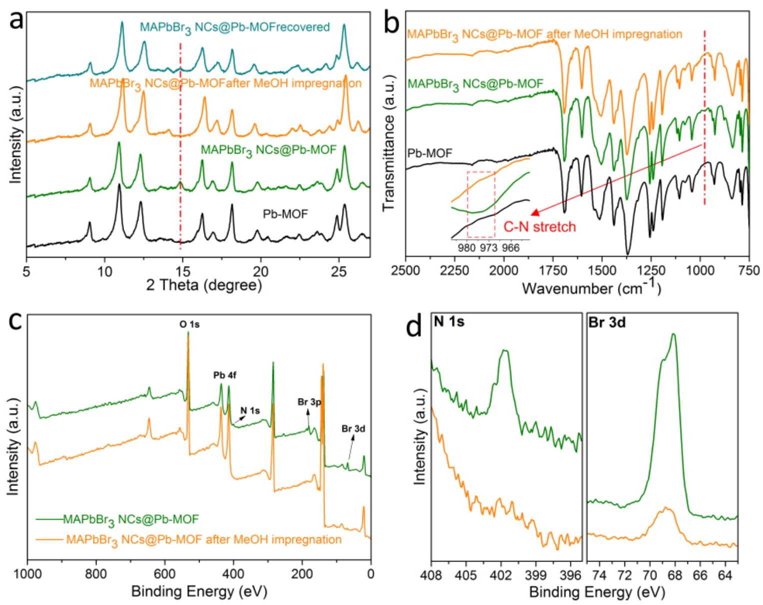

Supplementary Figure 20 XRD (a), FTIR (b) and XPS (c, d) characterization of the $MAPbBr_3$ NCs@Pb-MOF before and after methanol impregnation treatment.

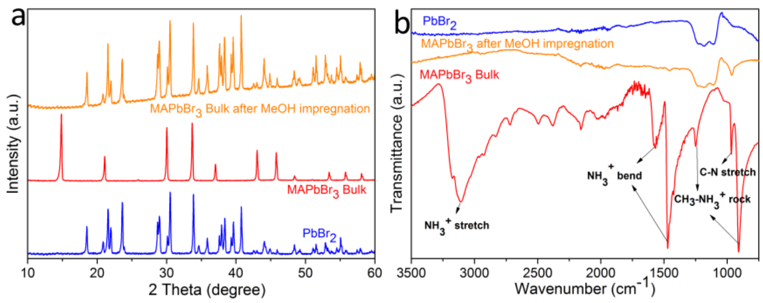

Supplementary Figure 21 XRD pattern (a) and FTIR spectra (b) of the $MAPbBr_3$ bulk before and after methanol impregnation treatment.

In order to further figure out the degradation pathway, we directly did the methanol impregnation experiment of $MAPbBr_3$ bulk sample to avoid the influence of the MOF matrix (the Pb-MOF does not change or degrade in methanol). Obviously, after methanol impregnation, the crystal structure of $MAPbBr_3$ has been changed to $PbBr_2$ (Supplementary Fig. 21a). Moreover, these C, N-containing groups have almost disappeared (Supplementary Fig. 21b). Therefore, we considered that after methanol impregnation, the $MAPbBr_3$ NCs in Pb-MOF have been mainly degraded to $PbBr_2$ and other C, N-containing organic constituents which can be easily washed away because of their good solubility (in methanol) and volatility.

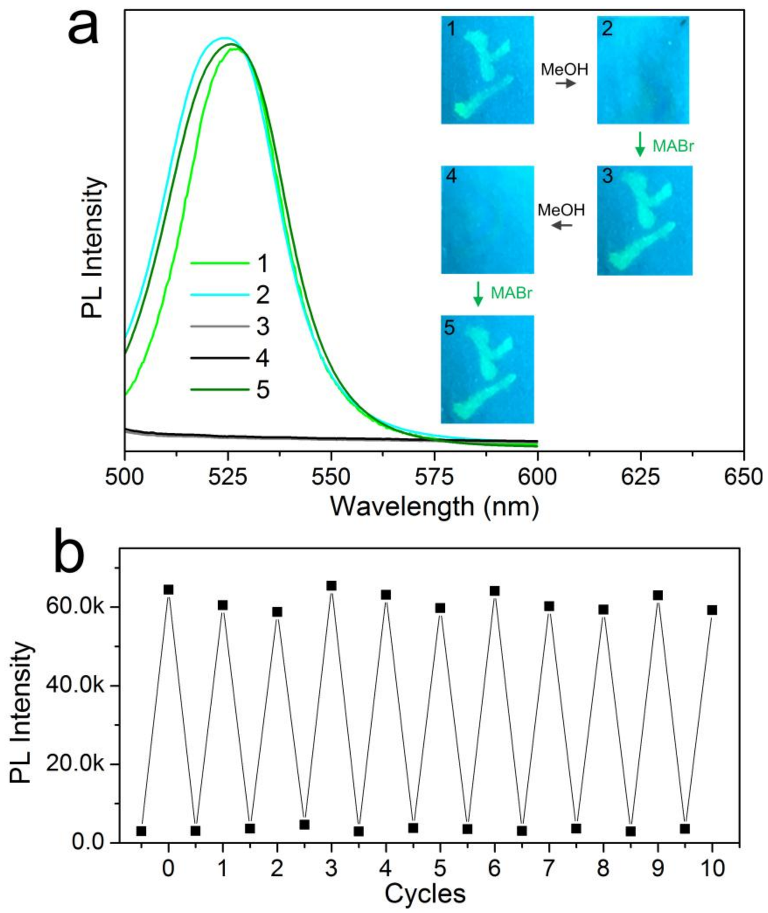

Supplementary Figure 22 Reversible fluorescence switching of the $MAPbBr_3$ NCs@Pb-MOF pattern. (a) Sequential optical images and PL emission spectra of $MAPbBr_3$ NCs@Pb-MOF after two cycles of impregnation-recovery process under ambient light. 1, 2, 3, 4, 5 represent the original, impregnated, recovered, secondary impregnated and secondary recovered $MAPbBr_3$ NCs@Pb-MOF pattern respectively. (methanol impregnation for encryption and MABr spraying for decryption); (b) PL intensity of $MAPbBr_3$ NCs@Pb-MOF as a function of cycle number.

Supplementary Table 1 Element contents of the $MAPbBr_3$ NCs@Pb-MOF sample from the XRF data.

Sample	*Element	Wt %	At %	#Calculated percentage of $MAPbBr_3$ NCs/Pb-MOF %	Average
1	Pb	47.75	5.49	2.73	3.15
	C	37.4	74.24		
	O	13.31	19.82		
	Br	1.51	0.45		
2	Pb	49.75	5.94	3.76	
	C	36.95	76.21		
	O	11.1	17.17		
	Br	2.16	0.67		
3	Pb	59.02	8.36	2.95	
	C	31.91	78.05		
	O	7	12.84		
	Br	2.03	0.74		

* Due to the light weight and the small amount of the N element in our sample, the content of this element has not been detected.

# The percentage of the perovskite NCs in MOF matrix are estimated by the atomic percent of Pb and Br.

Supplementary Table 2 Element contents of the $MAPbBr_3$ NCs@Pb-MOF sample from the XPS data.

Sample	Elements	Pos.	FWHM	Area	At %	#Calculated percentage of $MAPbBr_3$ NCs/Pb-MOF %	Average
1	C 1s	284.84	1.66	5446.29	62.37	19.07	20.88
	N 1s	398.80	1.84	239.43	1.60		
	O 1s	528.60	2.57	6586.57	26.88		
	Pb 4f	136.00	1.61	15236.19	5.82		
	Br 3p	65.70	2.09	1108.65	3.33		
2	C 1s	284.80	2.60	8175.19	65.78	20.18	
	N 1s	401.30	1.91	267.69	1.26		
	O 1s	532.70	3.99	8652.08	24.81		
	Pb 4f	138.50	2.09	18893.46	5.07		
	Br 3p	68.80	2.61	1449.93	3.07		
3	C 1s	284.80	1.50	9729.56	69.69	23.40	
	N 1s	401.90	1.61	790.77	2.06		
	O 1s	531.40	2.27	7949.96	20.71		
	Pb 4f	138.70	1.34	18549.00	4.43		
	Br 3p	68.20	1.96	1654.23	3.11		

The percentage of the perovskite NCs in MOF matrix are estimated by the atomic percent of Pb and Br.
